# RNA Não Codificante Longo, Apoptose e Cardiotoxicidade Induzida por Doxorrubicina

**DOI:** 10.36660/abc.20240331

**Published:** 2024-07-30

**Authors:** Carolina R. Tonon, Bertha F. Polegato

**Affiliations:** 1 Universidade Estadual Paulista Departamento de Clínica Médica Faculdade de Medicina de Botucatu Botucatu SP Brasil Faculdade de Medicina de Botucatu - Departamento de Clínica Médica - Universidade Estadual Paulista (UNESP), Botucatu, SP – Brasil

**Keywords:** Doxorrubicina/toxicidade, Cardiotoxicidade, Insuficiência Cardíaca, Estresse Oxidativo, Fator de Indução de Apoptose, RNA longo não codificante

A doxorrubicina é um dos medicamentos quimioterápicos mais eficazes utilizados no tratamento de muitos tipos de malignidades sólidas e hematológicas.^1^ No entanto, causa vários efeitos adversos. A cardiotoxicidade é o efeito colateral mais importante porque pode levar ao desenvolvimento de insuficiência cardíaca, uma condição crônica com altas taxas de mortalidade, com risco de 15-30% em 1 ano e risco de até 75% em 5 anos em populações específicas.^2^

A fisiopatologia da cardiotoxicidade induzida pela doxorrubicina não é completamente compreendida. Estão envolvidas vias clássicas, como dano direto ao DNA, estresse oxidativo, inflamação, alteração no trânsito de cálcio intracelular, inibição de genes relacionados a proteínas musculares, disfunção mitocondrial e ativação de vias de morte celular, como a apoptose.^3^ No entanto, novos mecanismos ganharam mais importância na última década, principalmente na área genética.

No século XX, acreditávamos que a parte mais importante do genoma era a codificação das proteínas. As sequências genéticas não relacionadas à codificação de proteínas, geralmente, recebiam menor atenção e eram chamadas de RNA não codificante. Nas primeiras décadas do século XXI, descobrimos que algumas pequenas sequências de RNA não codificante poderiam atuar como fatores transcricionais, pós-transcricionais e translacionais, interferindo e regulando a expressão proteica.^4^ No entanto, os genes de codificação de proteínas permaneceram em destaque. Mais recentemente, com os avanços na biologia molecular e na transcriptômica, descobrimos que o nosso genoma é maioritariamente transcrito em RNA mais longos sem capacidade de codificação de proteínas (lncRNA). Isso mudou o paradigma vigente e nos alertou que deveríamos começar a pensar de forma diferente sobre a expressão gênica.^4^ Nesse contexto, o número de estudos sobre o papel do lncRNA tem aumentado rapidamente. Essas sequências de RNA têm mostrado associação com doenças cardiovasculares, estando envolvidas no estresse oxidativo, apoptose e outras vias.^5^ Além disso, a perda de um lncRNA específico (OXCT1-AS1) no tecido cardíaco resultou na diminuição do desenvolvimento da força contrátil.^6^ No entanto, a função exata dos lncRNAs permanece desconhecida.

Nesta edição dos Arquivos Brasileiros de Cardiologia, Chen et al.,^7^ apresentaram extensa pesquisa sobre o papel do lncRNA OXCT1-AS1 na apoptose miocárdica induzida pela doxorrubicina em modelo de cultura de células de miócitos. Cardiomiócitos humanos AC16 foram cultivados e tratados com 5 μM de doxorrubicina por diferentes períodos para induzir lesão celular miocárdica. O tratamento com doxorrubicina prejudicou a viabilidade das células e o nível de expressão de lncRNA OXCT1-AS1 diminuiu de forma dependente do tempo após o tratamento. Esses achados foram associados ao aumento da apoptose celular, caracterizada por diminuição da expressão de Bcl-2, uma proteína anti-apoptótica, e aumento da expressão de proteínas pró-apoptóticas Bax, caspase 3 clivada e caspase-9 clivada. Por outro lado, a superexpressão do lncRNA OXCT1-AS1 melhorou a viabilidade das células e reduziu a apoptose sob estimulação com doxorrubicina.

Para determinar os mecanismos pelos quais OXCT1-AS1 afetou a apoptose de células AC16, os autores identificaram que o tratamento com doxorrubicina aumentou de forma dependente do tempo a expressão de miR-874-3p e reduziu a expressão dos genes RGS4, BDH1, HEG1 nas células. A superexpressão de OXCT1-AS1 diminuiu significativamente a expressão de miR-874-3p e aumentou a expressão de BDH1. Além disso, a superexpressão de BDH1 preservou a viabilidade celular e reduziu a apoptose causada pela doxorrubicina.

Os dados permitiram aos autores levantarem a hipótese de que a superexpressão de OXCT1-AS1 poderia aumentar a viabilidade dos cardiomiócitos e suprimir a apoptose induzida por doxorrubicina por meio da interação com a expressão do miR-874-3p e BDH1.

Apesar dos resultados interessantes, precisamos considerar que este é um resultado preliminar sobre o lncRNA OXCT1-AS1. O presente estudo foi realizado apenas in vitro. Considerando que o lncRNA pode participar de muitos processos fisiológicos (diferenciação celular, desenvolvimento celular, resposta inflamatória, vias de transporte celular, metabolismo de glicose e lipídios e produção de hormônios),^4^ experimentos in vivo serão necessários para confirmar o papel do lncRNA OXCT1-AS1 em organismos complexos.

Além disso, o lncRNA OXCT1-AS1 poderia desenvolver outras funções no contexto do tratamento do câncer. Por exemplo, a regulação positiva de lncRNA induz metástases em câncer de pulmão não pequena células em modelos in vitro e in vivo^8^ e promove a proliferação e invasão de células cancerígenas da bexiga.^9^ Além disso, o lncRNA OXCT1-AS1 é regulado positivamente no glioblastoma, prevendo pior prognóstico, e o knockdown do lncRNA OXCT1-AS1 atenuou a gravidade do glioma in vivo.^10^

O presente estudo^7^ destacou a importância da pesquisa realizada na área de lncRNA para melhor compreender os processos relacionados a essas moléculas ainda pouco estudadas e nos permite avançar alguns passos em nosso conhecimento científico.
