# Supplementary material for: RNA Não Codificante Longo, Apoptose e Cardiotoxicidade Induzida por Doxorrubicina
Source: Arq Bras Cardiol. 2024 Jul 30;121(6):e20240331. [Article in Portuguese] doi: 10.36660/abc.20240331 (PMC11364448; doi:10.36660/abc.20240331)
Supplement: Translation

# Translation: Long Non-Coding RNA, Apoptosis, and Doxorubicin-Induced Cardiotoxicity

**DOI:** 10.36660/abc.20240331i

**Authors:** Carolina R. Tonon, Bertha F. Polegato

**Affiliations:** 1 Botucatu SP Brazil Faculdade de Medicina de Botucatu - Departamento de Clínica Médica - Universidade Estadual Paulista (UNESP), Botucatu, SP – Brazil

**Keywords:** Doxorubicin/toxicity, Cardiotoxicity, Heart Failure, Oxidative Stress, Apoptosis Inducing Factor, RNA, Long Noncoding

Doxorubicin is one of the most effective chemotherapy drugs used in the treatment of many types of solid and hematologic malignancies.^1^ However, it causes several adverse effects. Cardiotoxicity is the most important collateral effect because it can lead to the development of heart failure, a chronic condition with high mortality, reaching with a 1-year risk of 15-30% and a 5-year risk of up to 75% in specific populations.^2^

The pathophysiology of doxorubicin-induced cardiotoxicity is not completely understood. Classical pathways are involved such as direct damage to DNA, oxidative stress, inflammation, alteration in intracellular calcium transient, inhibition of muscle protein-related genes, mitochondrial dysfunction, and activation of cell death pathways, such as apoptosis.^3^ However, new mechanisms have gained more importance in the last decade, mainly in the genetic field.

In the 20^th^ century, we believed that the most important part of the genome was the codification of proteins. The genetic sequences not related to protein coding usually received less attention and were called non-coding RNA. In the first decades of the 21^st^ century, we discovered that some little sequences of non-coding RNA could act as transcriptional, post-transcriptional, and translational factors, interfering with and regulating protein expression.^4^ However, the protein codification genes remained in the spotlight. More recently, with the advances in molecular biology and transcriptomics, we have discovered that our genome is mostly transcribed into longer RNAs with no protein-coding ability (lncRNA). It changes our paradigm and we should start to think differently about gene expression.^4^ In this sense, the number of studies about the role of lncRNA has increased rapidly. These RNA sequences have shown an association with cardiovascular diseases, being involved in oxidative stress, apoptosis, and other pathways.^5^ Moreover, the loss of a specific lncRNA (OXCT1-AS1) in heart tissue resulted in decreased contractile force development.^6^ Nevertheless, the exact function of lncRNAs remains unknown.

In this issue of *Arquivos Brasileiros de Cardiologia*, Chen et al.^7^ presented extensive research on the role of lncRNA OXCT1-AS1 in myocardial apoptosis induced by doxorubicin in a myocyte cell culture model. Human AC16 cardiomyocytes were cultured and treated with 5 μM of doxorubicin for different periods to induce myocardial cell injury. Doxorubicin impaired the viability of cells and the expression level of lncRNA OXCT1-AS1 was time-dependently decreased after the treatment. These findings were associated with increased cell apoptosis combined with decreased expression of Bcl-2, an anti-apoptotic protein, and increased expression of pro-apoptotic proteins Bax, cleaved caspase 3, and cleaved caspase-9. On the other hand, the overexpression of lncRNA OXCT1-AS1 improved the viability of cells and reduced apoptosis under doxorubicin stimulation.

To determine the mechanisms by which OXCT1-AS1 affected AC16 cell apoptosis under doxorubicin stimulation, the authors identified that doxorubicin treatment time-dependently increased miR-874-3p expression and reduced the expression of RGS4, BDH1, HEG1 genes in the cells. The overexpression of OXCT1-AS1 significantly suppressed the expression of miR-874-3p and enhanced the expression level of BDH1. Additionally, the overexpression of BDH1 reversed AC16 cell viability and reduced apoptosis caused by doxorubicin.

The data allowed the authors to hypothesize that overexpressing OXCT1-AS1 could enhance cardiomyocyte viability and suppress cardiomyocyte apoptosis under doxorubicin stimulation, through interaction with miR-874-3p and BDH1 expression.

Despite the interesting results, we need to keep in mind that this is a preliminary result about lncRNA OXCT1-AS1. The present study was conducted only *in vitro*. Considering that lncRNA can participate in many physiological processes (cell differentiation, cell development, inflammatory response, cellular transport pathways, glucose, and lipid metabolism, and hormone production),^4^ *in vivo* experiments will be needed to confirm the role of lncRNA OXCT1-AS1 in complex organisms.

Additionally, lncRNA OXCT1-AS1 could develop other roles in the context of cancer treatment. For instance, upregulation of lncRNA induces metastasis in non-small-cell lung cancer *in vitro* and *in vivo* ^8^ and can promote bladder cancer cell proliferation and invasion.^9^ Also, lnc-RNA OXCT1-AS1 is upregulated in glioblastoma, predicting a poorer prognosis, and the knockdown of lncRNA OXCT1-AS1 attenuated the severity of glioma *in vivo.*^10^

The present study^7^ highlighted the importance of the research conducted in the field of lncRNA to better understand the processes related to these poorly understood molecules and allows us to progress some steps in our scientific knowledge.
